# Annual Long-Distance Migration Strategies and Home Range of Chinese Sparrowhawk (*Accipiter soloensis*) from South China

**DOI:** 10.3390/ani11082237

**Published:** 2021-07-29

**Authors:** Xiao Min, Zijing Gao, Yuanfeng Lin, Chang-Hu Lu

**Affiliations:** Department of Zoology, College of Biology and the Environment, Nanjing Forestry University, Nanjing 210037, China; mx1543745024@163.com (X.M.); gzj@njfu.edu.cn (Z.G.); linyuanfeng@njfu.edu.cn (Y.L.)

**Keywords:** raptor, *Accipiter soloensis*, migration strategies, stop-overs, home range, Chinese sparrowhawk, GPS tracking

## Abstract

**Simple Summary:**

The migration strategies and activity patterns of the Chinese Sparrowhawk, a small raptor species, remain unknown. Long-distance migratory birds have the habit of using stop-over sites during migration in order to help them supplement energy and restore their physical strength. We put GPS satellite positioning devices on Chinese Sparrowhawks, with the objective of determining when they start their migration, the route, whether they stopped during migration, habitat preferences, and where they ultimately wintered. This was conducted to help us understand the entire migration strategy of the Chinese Sparrowhawk. At the same time, based on satellite location information, we constructed a comprehensive picture of its summer, winter, and stop-over home ranges, and explored the associated activity patterns.

**Abstract:**

From 2018 to 2019, two Chinese Sparrowhawks (Bird 01, male; Bird 02, female), *Accipiter soloensis*, were captured and fitted with Global Positioning System (GPS) loggers in order to identify summering and wintering sites, migration routes, and stop-over sites. The Chinese Sparrowhawks were first fitted with backpack solar GPS satellite trackers in China in order to explore their migration routes. The two Chinese Sparrowhawks successfully completed their migration from southern China, through Nanning city of Guangxi province, China, to Vietnam, Laos, Myanmar, Thailand, Malaysia, and Singapore and finally arriving in Indonesia, where they stayed until the March of the following year. They then returned to China along the original route, arriving in Changsha city, Hunan province, China. The two individuals traveled more than 4000–5000 km. For the first time, telemetry data demonstrate, the linkages between their Indonesia wintering sites, their stop-over sites in Southeast Asia, and their breeding/summering sites near south Yangtze River in the south-central part of China. During this long-distance migration, 2653 bird satellite sites were received. The autumn migration durations for the two Chinese Sparrowhawks were 84 days and 50 days, respectively, compared to 83 days and 49 days in spring. The median stop-over duration was 12.7 and 9.3 days, respectively and the median speed of travel was 74.2 km/day during the autumn migration and 73.9 km/day during the spring migration. Furthermore, two and one stop-over sites and one and three stop-over sites were used during the autumn and spring migrations of Chinese Sparrowhawks 01 and 02, respectively. The Chinese Sparrowhawks migrated long distances and used stop-over sites during their migration. Based on the home range analysis, we can conclude that Chinese Sparrowhawks reach their maximum home range in the summer and have multiple nuclear domains.

## 1. Introduction

Chinese Sparrowhawks are widely distributed in southern China, most of which are summer migratory birds. There are also a few summer resident birds in northern China, and some overwintering birds or resident birds in southern China and Hainan Island [1]. Often living in the relatively open forestland, mountains, forests, farmland, villages, and so on, they may form a small group (or, even, large groups) during their migration in the spring and autumn [2]. The Chinese Sparrowhawk is a small-sized raptor, whose breeding sites—although imprecisely known—are in China and Korea, and whose wintering sites are thought to be mainly in the Philippines and in eastern Indonesia [3]. Its global population is estimated to be six figures [4]. Very few records exist from their presumed wintering range, suggesting that the winter distribution remains largely unknown [5,6,7]. Recent migration research [8] has shown that at least 350,000 individuals of this species migrate to eastern Indonesia each autumn, through both the Sangihe Talaud Archipelago in the north and Bali in the west.

The species is less predatory, eating frogs, lizards, insects, and occasionally small birds and mice [9]. Chinese Sparrowhawks have been found on the Korean peninsula outside China and have been found to winter in the Philippines, Malaysia, Indonesia, and New Guinea [10]. They have also been recorded in the Indian subcontinent [11]. Chinese Sparrowhawks are few and very rare and have been listed in the second level of the China National Key Protected Wild Animals List.

Migration theory predicts that many migratory birds make long, uninterrupted flights over difficult terrain between their summering and wintering sites. Raptors migrate thousands of kilometers, stopping at multiple locations to replenish their energy [12]. GPS tracking on Eastern Imperial Eagles (*Aquila heliacal*) found that the maximum distance traveled in a single day was 130 km when the bird crossed the Dardanelles [13]. In the Arctic, scientists have used satellite tracking to follow 56 peregrine falcons (*Falco peregrinus*) from six populations; their results indicated that global warming will influence migration strategies and will diminish the breeding ranges of the peregrine populations of the Eurasian Arctic [14]. In studies of other bird species, such as the Purple Heron (*Ardea purpurea*), stop-overs are rarely used during migration [15]. Studies of Gray Heron (*Ardea cinerea*) migration have found that no stop-overs seem to be used during migration [16]. In general, many species are expected to make one or more stop-overs, in order to replenish their energy due to the higher flight energy consumption of birds [17]. However, the lack of suitable high-quality habitats along the migration route may force migrants to skip unfavorable sites [18]. Therefore, we used telemetry data to test whether Chinese Sparrowhawks exhibit similar strategies in sharing breeding and wintering sites.

On account of their conservation status, we still lack knowledge about the Chinese Sparrowhawk’s precise summering and wintering distributions, migration routes, home range, and critical stop-over sites in between routes. Previous studies on the species have focused on its reproductive ecology and the genetic diversity of the population, while little information is available regarding its ecology after reproduction. For this reason, we deployed the Global Positioning System/Global System for Mobile Communications (GPS/GSM) loggers on Chinese Sparrowhawks in Changsha, Hunan province, China in order to confirm the connection between summering and wintering sites as well as to locate potentially important migration stop-over sites. We calculated their home range in three periods (summering, wintering, and stop-over sites) and compared the differences between these three periods.

## 2. Materials and Methods

### 2.1. Capture of Individuals and Transmitter Attachment

To understand the migration strategies of the Chinese Sparrowhawk, we used global positioning system (GPS) satellite trackers. In 2018–2019, we attached GPS satellite trackers to six adult Chinese Sparrowhawks caught in nets on the outskirts of Changsha, Hunan province, China. A total of four of them lost tracking signals for unknown reasons and, so, this paper only considers the two Chinese Sparrowhawks (Table 1) who successfully completed their annual migration. Through the color of their iris, we found that Bird 01 was male (red and black iris), while Bird 02 was female (yellow iris). The travel speed (km/h) of the birds was calculated by dividing the distance between two consecutive positions by the time period. We defined the beginning of migration as the day a bird flew 10 km (in any direction) away from its habitat [19]. The end of migration was defined as the day when the bird flew only a short distance north/south (less than 10 km per day) for more than 5 days. If the speed of the bird became more than 10 km/h between two positions during migration, it was migrating [20].

We used the HQ series of miniature GPS trackers for wildlife produced by Hunan Xin Shi global co., LTD. The device equipped with solar cells was fixed on the back of the birds, according to the method of backpack installation. The weight of this model was less than 3% of the body weight of the Chinese Sparrowhawk [21]. The weight of Bird 01 was 120.3 g, and that of Bird 02 was 124.5 g. The tracker recorded the geographic coordinates of the bird’s location through the GPS satellite positioning system. Within 48 hours after installation and starting up the device, the satellite site data were transmitted back in real-time (at 1 h intervals), and, after 48 h, the data were transmitted back to every 5 sites. The GPS trackers used in the study were designed to transmit data using roaming services once the birds left the country, sending data every 48 h. If there was no mobile network coverage around, the recorded location was stored in the GPS tracker memory until the next time the device started searching for a mobile network connection 48 h later. If there was no mobile network coverage, the device could store up to 12,000 loci. The GPS tracker used the wgs-84 geodetic coordinate system, and the positioning accuracy of the device adopted the following linear regression analysis method: Error = 2.679243 × HD0P + 0.59144. In addition to the latitude and longitude of the birds, the GPS tracker also provided activity data on their flight speed, altitude, heading, temperature, and movement [22]. As the environment is constantly changing, the temperature measured by our equipment combined the ambient temperature and body temperature.

### 2.2. Identifying Stop-Over Sites 

According to the migration conditions, raptor migration can be divided into the breeding season, the autumn migration period, the overwintering period, and the spring migration period. In this study, the method used to analyze changes in migration period was adopted to determine the “date threshold” of the changes in migration state [23]. It was considered that significant changes in longitude, latitude, and activity distance occurred at a certain time, which comprised the critical points of the changes in migration state [24]. If the data before and after the critical point were incomplete, the intermediate value of the adjacent date was taken. If the tracker did not record arrival and departure dates, it could be assumed to be the intermediate date between the point before and after arrival and departure. Tracking points with a speed of less than 10 km/h during migration were selected from ArcGIS 10.1. According to the distribution of the sites, regions with a radius of 25 km and at least two sites with different dates were defined as migration resting places [25].

### 2.3. Defining Migration Parameters

The migration duration was defined as the total duration of spring and autumn migrations, including the periods spent at stop-over sites. The number of stop-over sites throughout each migration period was calculated using the criteria above in order to define how many times the Chinese Sparrowhawks used separate stop-over sites, and the stop-over duration was derived as the cumulative sum of all of the days spent at all of these stop-over sites during each migration episode. We calculated the migration distance by summing the distances of all migration legs in the spring/autumn migrations. The distance of each migration leg was calculated by summing the distance of the consecutive locations of the migration leg rather than by simply connecting the starting and ending points of the migration leg. We marked the linear distance as the straight-line distance from the summering site point to the wintering site point. The number of travel days was defined as the total duration of migration minus stop-over days [26].

### 2.4. Statistical Analysis

According to the GPS satellite migration data of the system, ArcGIS V10. 1, Excel 2007, and Google Earth were used to analyze the migration route of the Chinese Sparrowhawks from 2018 to 2019. Home ranges were calculated using the ArcGIS software (ESRI, Redlands, CA, USA) in conjunction with the Hawth’s Analysis Tools for ArcGIS extension and the Biotas 2.0a (Ecological software solution^TM^, UT, USA) for the analysis module. We used adaptive kernels to calculate the 100% MCP (home range), and 95%, 75%, and 50% (core area) FK contours [27].

## 3. Results

Overall, we obtained complete spring and autumn migration tracks from the two individuals based on the GPS satellite data; that is, we obtained two autumn and two spring migration tracks from two different individuals.

### 3.1. Migration Routes

**Figure 1:**
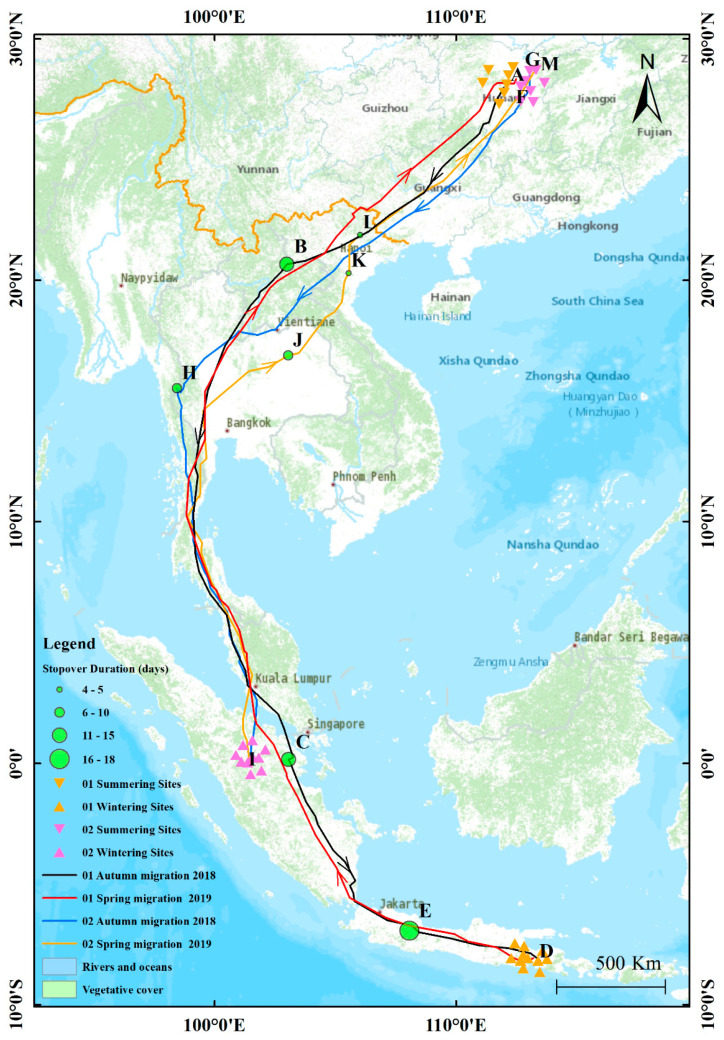
Migration routes and main sites used by individual Chinese Sparrowhawks linked by solid lines. Green circles indicate stop-over sites. Summering sites: A, F, G, M; Wintering sites: D, I; Stop-over sites: B, C, E, H, J, K, L.

We successfully tracked the annual migration of Chinese Sparrowhawks 01 and 02 (Figure 1; Table 1). Chinese Sparrowhawk 01 was released from 21 May 2018 to 8 September 2019 in Changsha, Hunan province, China. It took 476 days to receive 964 pieces of satellite positioning data. According to the satellite positioning information, Chinese Sparrowhawk 01 started its southward migration on 2 September 2018, which was defined as the beginning date of the bird’s autumn migration. It lasted 84 days and arrived near the south of Argopuro, Indonesia (113°29′29″ E, 8°10′17″ S) on 24 November 2018. It stayed there until 1 March 2019, when it started its northward migration, which was defined as the beginning date of the bird’s spring migration. It lasted 83 days, and the bird migrated to near Changsha city, Hunan province, China on 22 May 2019 (112°29′57″ E, 28°20′44″ N), completing the entirety of its autumn and spring migrations. The bird’s GPS satellite tracker remained operational until 8 September 2019.

Chinese Sparrowhawk 02 was released from 22 May 2018 to 18 September 2019 in Changsha, Hunan province, China, taking a total of 485 days to receive 879 pieces of satellite positioning data. According to the satellite positioning information, Chinese Sparrowhawk 02 started its southward migration on 3 September 2018, which we defined as the beginning date of the bird’s autumn migration. It lasted 50 days, and the bird arrived in the National Park of Tesso Nilo, in central Indonesia (101°25′19″ E, 0°7′46″ S) on 23 October 2018. It stayed there until 13 March 2019, when it started its northward migration, which we defined as the beginning date of the bird’s spring migration. It lasted 49 days, and the bird migrated to the north of Changsha city, Hunan province, China (113°15′8″ E, 28°42′34″ N) on 30 April 2019, completing the entirety of its autumn and spring migrations. The bird’s GPS satellite tracker remained operational until 18 September 2019.

Both Chinese Sparrowhawks 01 and 02 experienced a long-distance journey across eight countries, taking a long time, and both birds used stop-over places for food supplementation and rest during their migration. According to their locations on Google maps, the selected stop-over sites were mostly in dense forestland, close to water sources, away from human activity, and near mountains. The length of stay varied, depending on flying distance and weather factors.

### 3.2. Summering Sites, Wintering Sites and Stop-Over Sites

Both individuals lived in suburbs far from the city, with dense forests near rivers, and moved to multiple locations, with an average stay of 97 days. After three months of breeding, their journey to the south began. In the autumn migration route, Chinese Sparrowhawk 01 used two stop-over sites for rest and supply, while Chinese Sparrowhawk 02 used one stop-over site for rest and supply. Chinese Sparrowhawk 01 stayed in Laos and Indonesia for 14 and 15 days, respectively. Chinese Sparrowhawk 02 stayed in Burma for 9 days. In the end, the two Chinese Sparrowhawks arrived in Indonesia for the winter, which lasted for 98 and 142 days, respectively. The two Chinese Sparrowhawks wintered in different locations in Indonesia (see Table 2). Bird 01 chose the further east province of East Java, while Bird 02 chose the Riau province near Singapore in Indonesia, both of which are heavily forested.

From March 2019 (Table 2), the two Chinese Sparrowhawks embarked on their spring migration, with Bird 01 and 02 using one and three stop-over sites, respectively. Bird 01 spent 18 days in Malang, Indonesia, while Bird 02 stopped at one stop-over site in Thailand and two stop-over sites in Vietnam, for 10, 4, and 5 days, respectively. The birds eventually arrived in Changsha, Hunan Province, China, where they stayed for the summer of 2019. Birds 01 and 02 stayed for 110 and 124 days, respectively. From the research, we can see that the Chinese Sparrowhawks summered and wintered for more than 3 and 4 months, respectively.

### 3.3. Migration Strategies

Chinese Sparrowhawks migrate from high latitudes to low latitudes in the autumn and from low latitudes to high latitudes in the spring of the following year. From the results, it can be seen that Bird 01 began to gradually migrate southward from the beginning of September 2018, accelerated southward from the end of September to November 2018, reached around 10° south latitude at the end of November, stayed until the end of March of the next year, then began to migrate northwards, which lasted until the end of May, and reached the breeding ground. Bird 02 began to migrate south from early September 2018, which lasted until the end of October, reached the equator, and stayed until the end of March of the next year, and then started to move north. The northward migration process lasted until the end of April.

It can be seen that there were periodic fluctuations in flight height during the breeding season, reflecting the activities of the individual birds in foraging and seeking mates. Their temperature peaked during the breeding season, and we inferred that a large number of flight movements during the breeding season led to this increase. During migration, the flight altitude showed a huge drop, reflecting the need to fly over mountains and to follow the airflow during the long migration. They flew at a maximum altitude of 1800 m. The smallest altitude was when they dropped over wintering sites, reflecting their low activity. We hypothesized that by reducing the amount of activity, they could store enough energy for the spring migration in order to reach the breeding sites early. The temperature fluctuation during the migration period and the overwintering period was not large, but it fluctuated the most in the breeding season, reflecting the frequent and abundant activities occurring in the breeding season.

The two individuals had a difference of one day between spring and autumn migrations, and there was no obvious seasonal contrast. However, Bird 01 took 34 days longer than Bird 02 in terms of migration time, and we deduced that the migration time was longer due to the fact that Bird 01 moved farther from the wintering site than Bird 02 (i.e., to the easternmost part of Indonesia).

### 3.4. Home Range

We calculated the home range of two Chinese Sparrowhawks in their summering sites, wintering sites, and stop-over sites during their migration from May 2018 to September 2019 (Table 3). Overall, the results of a minimum convex polygon (100% MCP) showed that the home range of the Chinese Sparrowhawk was 0.0440–6730.9275 km^2^. The results of the fixed kernel (FK) showed that the home range of the Chinese Sparrowhawk was 0.0096–36.5494 km^2^, 0.0021–14.5752 km^2^, or 0.0006–7.7079 km^2^. According to the calculation of the minimum convex polygon (100% MCP) method, the home range of the Chinese Sparrowhawk in the summering sites was 415.2083–6730.9275 km^2^ (mean ± SD = 2557.9543 ± 2965.0585 km^2^); in the wintering sites, it was 7.8153–9.7193 km^2^ (mean ± SD = 8.7673 ± 1.3463 km^2^); and in stop-over sites during migration, it was 0.0440–0.4575 km^2^ (mean ± SD = 0.2311 ± 0.1537 km^2^). According to the fixed kernel calculation (50% or core area FK), the home range of the Chinese Sparrowhawks in the summering sites was 0.3747–7.7079 km^2^ (mean ± SD = 3.0473 ± 3.2412 km^2^); in the wintering sites, it was 0.1680–0.3892 km^2^ (mean ± SD = 0.2786 ± 0.1564 km^2^); and in the stop-over sites during migration, it was 0.0006–0.0202 km^2^ (mean ± SD = 0.0087 ± 0.0086 km^2^). The home range of the Chinese Sparrowhawk in different periods showed extremely significant differences (Chi-square test, *p* = 0.007, 0.009). There was no significant difference of the home range in the stop-over sites between the autumn and spring migrations (Mann–Whitney U-test, *p* = 0.724, 0.289, 0.077, 0.157, 0.480; Table 4). In the 100% MCP and 95%, 75%, and 50% FK home range map, the number of nuclear domains of the Chinese Sparrowhawk at the summering sites was significantly higher than that in the wintering sites and the stop-over sites in the migration period. The range of activities in the summer was larger, with multiple core areas.

## 4. Discussion

For the first time, we studied the migration route and migration strategies from summering sites to wintering sites of the Chinese Sparrowhawk, based on GPS satellite data. The migration routes of the two Chinese Sparrowhawks determined the relationships among their stop-over sites, summering sites, and wintering sites [28]. During the spring and autumn migrations, a total of seven stop-over sites were used, mainly in South and Southeast Asia, and were distributed in large forests. These sites are important for the migration of the Chinese Sparrowhawk population [29]. During migration, Chinese Sparrowhawk 01 reached the highest fly height of 1804 m, and Chinese Sparrowhawk 02 reached the highest fly height of 1673 m, which was relatively low, in the summer breeding season and overwintering period, respectively. During migration, due to the use of air flow flight to save energy when crossing mountains, their flight altitude was relatively high [30]. During such a long migration, the number of stop-overs was very small; they only took one day to fly across the Strait of Malacca. Therefore, we believe that the Chinese Sparrowhawk has a very strong migration ability.

Some studies have shown that Chinese Sparrowhawks winter in Papua (Indonesian New Guinea) [31]. Our satellite tracking studies confirm this conclusion. By using fewer stop-over sites during the migration process, we think that the Chinese Sparrowhawk can gain enough energy before migration in order to reduce the rate of using such stop-over sites during migration and, as a result, shorten the time required to arrive at the summering and wintering sites ahead of time to find a suitable habitat and to look for a mate, respectively. Based on the use of GPS satellite trackers, we can clearly understand and describe the migration path of the Chinese Sparrowhawk.

Many migratory birds make long and non-stop flights between their summering and wintering sites over difficult terrain, including the Pacific Ocean, the Himalayas, and deserts [32]. Each of the two Chinese Sparrowhawks used only one stop-over during their one-way migration, indicating that they could complete a migration of more than 5000 km with only one stop-over, meaning that they could accumulate all of the necessary energy reserves with very few stop-overs [33]. In Indonesia, forests in monsoon season, secondary forests, swamps, and savanna are ideal winter habitats [34].

Minimum convex polygon (MCP) and fixed kernel (FK) models have been widely used in the calculation of the home range of wild animals [35]. Among them, the calculation results of the FK model are stable and are not strongly affected by extreme points, allowing them to accurately describe the use characteristics of the internal space of the home range of the animals [36]. Although the MCP model has some shortcomings, such as ignoring the spatial use characteristics within the home range and being sensitive to the surrounding extreme points, it can intuitively describe the spatial distribution characteristics of the home range and can be compared to the estimation results of other home range models, such that its calculation results are still of significance [37]. The summer breeding season is the time when the home range area is the largest, and there are multiple nuclear domains within the region, as the animals may expand their range and move back and forth between multiple core regions for mating and breeding purposes. In the overwintering period, the range of activity is relatively concentrated, the area of home range is significantly smaller than that of summer breeding period, and there are no multiple core areas. By contrast, the limited home range in winter may be a strategy to reduce fat consumption [38]. There was no significant difference in stop-over sites between spring and autumn migrations. As it is only a short stop-over for energy and rest, the activity area was much smaller than that of the summer breeding season and overwintering season. There were multiple nuclear domains, which we hypothesized were to increase the chances of finding food and continuing to migrate, aside from replenishing energy [39,40].

This research highlighted the precise nature of the migration strategies of Chinese Sparrowhawk during their spring and autumn migrations. We remain cautious of inferring too much from just two individuals. The process of the Chinese Sparrowhawks that are flying from China to Indonesia to winter is still far from clear but, based on our study of two individuals, we know that they still return to the same sites for the summer breeding season. As one of the smaller raptor species, we recommend further fieldwork and telemetry studies in order to confirm these strategies and to enhance our understanding of the contributions of different spring and autumn flyways of the Chinese Sparrowhawk as well as to improve the monitoring of their abundance and demographic parameters, which contributes to the annual rate of population change in this poorly known flyway.

## 5. Conclusions

Using GPS satellite tracking, our study revealed unprecedented details of the migratory route of the Chinese Sparrowhawk in Asia, especially in Indonesia (as a wintering site). However, due to the small sample size, we were unable to determine the summering and wintering sites of the entire species. Considering the importance of stop-overs in the annual cycle of migrating species, it is necessary not only to identify key stop-overs, but to also study the ecology of stop-overs in detail. Through home range calculation, it was concluded that the seasonal home range was significantly different. Wild animals increase their range of activities during the breeding season. In the summer breeding season, the home range of the Chinese Sparrowhawk reaches its maximum. Although the data were only obtained from two individuals, our study provides a detailed description of the migration strategies of the Chinese Sparrowhawk and its home range in different time periods. For further research, we need to track more individuals in order to understand important habitat characteristics, particularly within China, to support the enhanced conservation of this species.

## Figures and Tables

**Table 1:** Migration information of Chinese Sparrowhawks 01 and 02.

Bird Number	Bird 01 HQP589 21.05.2018		Bird 02 HQP593 22.05.2018	
Equipment Serial Number				
Release Date				
	Autumn Migration	Spring Migration	Autumn Migration	Springmigration
Start Date	02.09.2018	02.03.2019	03.09.2018	13.03.2019
Ending Date	24.11.2018	22.05.2019	23.10.2018	30.04.2019
Days of Migration (Days)	84	83	50	49
Number of GPS Sites	75	83	102	102
Migration Distance (km)	5550	5379	4110	4061
Linear Distance (km)	4103	4185	3470	3493
Minimum and Maximum Daily Distance (km)	36–212	32–252	18–198	16–262
Number of Stops	2	1	1	3
Stop-Over Duration (Days)	14, 15	18	9	10, 4, 5

**Table 2:** A date overview of the locations of Chinese Sparrowhawks 01 and 02 during the entire GPS satellite tracking.

Bird Number	Type of Sites	Site	Longitude and Latitude	Location	Country	Arrival–Departure
Bird 01	Summering sites	A	112°37′9″ E, 28°9′51″ N	Ningxiang	China	28.05.2018–02.09.2018
	Autumn migration stop-over sites	B	102°59′29″ E, 20°39′58″ N	Oudomxay	Laos	16.09.2018–29.09.2018
		C	103°3′19″ E, 0°9′3″ N	Riau	Indonesia	21.10.2018–04.11.2018
	Wintering sites	D	113°29′29″ E, 8°10′17″ S	Lawang	Indonesia	24.11.2018–01.03.2019
	Spring migration stop-over sites	E	112°49′16″ E, 8°14′57″ S	Malang	Indonesia	02.03.2019–19.03.2019
	Summering sites	F	112°42′22″ E, 28°11′24″ N	Ningxiang	China	22.05.2019–08.09.2019
Bird 02	Summering sites	G	113°1′54″ E, 28°31′4″ N	Changsha	China	28.05.2018–03.09.2018
	Autumn migration stop-over sites	H	98°26′7″ E, 15°31′54″ N	Thaton	Myanmar	27.09.2018–05.10.2018
	Wintering sites	I	101°25′19″ E, 0°1′2″ S	Sumatra	Indonesia	23.10.2018–13.03.2019
	Spring migration stop-over sites	J	103°2′12″ E, 16°52′44″ N	Non Sung	Thailand	30.03.2019–08.04.2019
		K	105°35′26″ E, 20°33′23″ N	Hanoi	Vietnam	14.04.2019–17.04.2019
		L	105°36′3″ E, 21°29′29″ N	Quang Minh	Vietnam	18.04.2019–22.04.2019
	Summering sites	M	113°15′26″ E, 28°42′31″ N	Wushi	China	01.05.2019–01.09.2019

**Table 3:** The results of home range of two Chinese Sparrowhawks in different location sites.

Bird Number	Season	Type of Site	Locations	100% Minimum Convex Polygon (km^2^)	Fixed Kernel (km^2^)		
					95%	75%	50% or Core Area
Bird 01	Autumn migration 2018	Summering sites	A	2617.2565	10.9326	3.6414	1.4716
		Stop-over sites	B	0.0592	0.0563	0.0171	0.0032
			C	0.1608	0.0867	0.0396	0.0196
		Wintering sites	D	9.7193	1.0919	0.3925	0.1680
	Spring migration 2019	Stop-over sites	E	0.3090	0.0096	0.0021	0.0006
		Summering sites	F	6730.9273	36.5494	14.5752	7.7079
Bird 02	Autumn migration 2018	Summering sites	G	468.4247	3.3148	0.9811	0.3747
		Stop-over sites	H	0.2338	0.1678	0.0473	0.0128
		Wintering sites	I	7.8153	2.0470	0.8079	0.3892
	Spring migration 2019	Stop-over sites	J	0.3535	0.0298	0.0081	0.0038
			K	0.0440	0.0847	0.0447	0.0202
			L	0.4575	0.0117	0.0026	0.0010
		Summering sites	M	415.2083	28.9693	8.5827	2.6349

**Table 4:** Statistical value based on Mann–Whitney U and Chi-square tests of home range of two Chinese Sparrowhawks.

	n		100% Minimum Convex Polygon (km^2^)	Fixed Kernel (km^2^)		
				**95%**	**75%**	**50% or Core Area**
Summering sites		Mean ± SD	2557.9543 ± 2965.0585	19.9415 ± 15.4373	6.9451 ± 5.9829	3.0473 ± 3.2412
	4	Max	6730.9275	36.5494	14.5752	7.7079
		Min	415.2083	3.3148	0.9811	0.3747
Wintering sites		Mean ± SD	8.7673 ± 1.3463	1.5695 ± 0.6754	0.6002 ± 0.2937	0.2786 ± 0.1564
	2	Max	9.7193	2.0470	0.8079	0.3892
		Min	7.8153	1.0919	0.3925	0.1680
Stop-over sites		Mean ± SD	0.2311 ± 0.1537	0.0638 ± 0.0557	0.0231 ± 0.0202	0.0087 ± 0.0086
	7	Max	0.4575	0.1678	0.0473	0.0202
		Min	0.0440	0.0096	0.0021	0.0006
Autumn migration stop-over sites		Mean ± SD	0.1513 ± 0.0877	0.1036 ± 0.0576	0.0347 ± 0.0157	0.0119 ± 0.0082
	3	Max	0.2338	0.1678	0.0473	0.0196
		Min	0.0592	0.0563	0.0171	0.0032
Spring migration Stop-over sites		Mean ± SD	0.2910 ± 0.1760	0.0340 ± 0.0350	0.0144 ± 0.0204	0.0064 ± 0.0093
	4	Max	0.4575	0.0847	0.0447	0.0202
		Min	0.0440	0.0096	0.0021	0.0006
Between the three type sites	13	*p*	0.007	0.007	0.007	0.009
Between the autumn and spring migration stop-over sites	7	*p*	0.289	0.077	0.157	0.480

Note: *p* < 0.05, significant difference; *p* < 0.01, extremely significant difference.

## Data Availability

None of the data were deposited in an official repository.
